# Impact of obesity on infarct size, circulating biomarkers, mitochondrial function and mortality in a Göttingen minipig myocardial infarct model

**DOI:** 10.1038/s41684-025-01533-4

**Published:** 2025-03-27

**Authors:** Mette Flethøj, Karina Poulsdóttir Debes, Cecilie Larsen, Caroline de Blanck, Trine Pagh Ludvigsen, Jeppe Kirchhoff, Jacob Eifer Møller, Steen Larsen, Jens P. Gøtze, Thomas Jespersen, Lisbeth Høier Olsen

**Affiliations:** 1 Research and Early Development, Global Drug Discovery, Novo Nordisk A/S, Måløv, Denmark; 2 Department of Veterinary and Animal Sciences, Faculty of Health and Medical Sciences, University of Copenhagen, Frederiksberg, Denmark; 3 Department of Biomedical Sciences, Faculty of Health and Medical Sciences, University of Copenhagen, Copenhagen, Denmark; 4 Department of Cardiology, Copenhagen University Hospital Denmark, University of Southern Denmark, Odense, Denmark; 5 Clinical Research Centre, Medical University of Bialystok, Bialystok, Poland; 6 Institute of Sports Medicine Copenhagen, Department of Orthopedic Surgery M, Copenhagen University Hospital – Bispebjerg and Frederiksberg, Copenhagen, Denmark; 7 Department of Clinical Biochemistry, Copenhagen University Hospital Denmark, Copenhagen, Denmark

**Keywords:** Myocardial infarction, Myocardial infarction

## Abstract

Obesity is a risk factor for the development of coronary artery disease and myocardial infarction (MI). However, most large animal studies of MI are performed in lean animals. Here we assessed the impact of obesity on echocardiographic findings, infarct size, circulating biomarkers, mitochondrial respiratory capacity and mortality in a closed-chest minipig model of MI. The initial study population consisted of 20 obese (median 60.0 kg [interquartile range 55.9–64.6 kg]) and 18 lean (25.0 kg [23.4–36.5 kg]) female Göttingen minipigs. The duration of obesity induction using a western-style diet was up to approximately 6 months (156 days [24–162 days]) before the induction of MI. The induction of MI by 120-min balloon occlusion of the left anterior descending coronary artery was feasible in 17 lean and 17 obese animals. Mortality was higher in obese compared with lean animals (53% versus 12%), driven primarily by refractory ventricular fibrillation during occlusion. Electrocardiographic findings showed longer QRS and QT intervals and more extensive ST-segment elevation in obese animals compared with lean animals during occlusion. Plasma concentrations of pro-atrial natriuretic peptide, pro-C-type natriuretic peptide and cardiac troponin T were significantly lower in obese compared with lean animals. Infarct size estimated 8 weeks after MI was significantly smaller in obese (10% [9–11%]) compared with lean animals (13% [13–15%]). Finally, mitochondrial-complex-I-linked respiratory capacity was overall significantly higher in obese animals; however, no group difference was found in intrinsic mitochondrial respiratory capacity.

## Abstract (web summary)

The study shows that, compared with lean animals, obesity significantly impacts echocardiographic findings, infarct size, circulating biomarkers, mitochondrial respiratory capacity and mortality in a minipig model of myocardial infarction.

## Main

Obesity is one of the major risk factors leading to coronary artery disease and has been associated with other lifestyle diseases, including hypertension and type 2 diabetes mellitus^1,2^. The prevalence of obesity has increased dramatically over recent decades and is a major factor contributing to the increased burden of cardiovascular disease^3,4^. Despite the predisposing effects in the development of acute myocardial infarction (MI), several studies have suggested that obesity may be linked to better survival once MI has occurred^5–7^. These studies investigated the outcome for patients admitted with acute MI and found an inverse association between body mass index (BMI) and all-cause mortality with higher survival rates in patients with obesity or overweight compared with normal-weight individuals^5,6,8^. The existence of such an obesity paradox is controversial and has been greatly debated^9,10^. Nevertheless, obesity has been shown to impact other aspects of cardiac physiology, including cardiac biomarkers and cardiac metabolism. Cardiac troponin T (cTnT) may be elevated in individuals with obesity and has been suggested to be indicative of subclinical myocardial injury^11^. Meanwhile, circulating levels of natriuretic peptides, especially atrial natriuretic peptide and brain natriuretic peptide, are known to be substantially lower in individuals with obesity, and this is thought to play a role in the development of systemic hypertension^12^. At the same time, natriuretic peptides are mediators with influence on fat metabolism and fat distribution, and their low levels in individuals with obesity may cause an unfavorable deposition of visceral and epicardial fat leading to cardiac dysfunction^13^. Cardiac metabolism is also directly affected by obesity, as studies have shown an increased reliance on fatty acid metabolism in the obese heart^14^. Thus, regardless of the existence of an obesity paradox, such an important risk factor should ideally be included in mechanistic and pathophysiological studies of coronary events.

Various animal models have been used to mimic acute MI^15,16^, and pigs are often preferred as large animal models owing to their similarity to human cardiac anatomy, physiology and metabolism, including similar coronary arterial structure and infarct sizes^17^. However, regardless of the importance of predisposing risk factors, only a few porcine MI studies have examined the impact of age, obesity, diabetes and hypertension^18^, and the majority of studies have used young, healthy, lean animals^19,20^. Therefore, the objective of the present study was to investigate the impact of obesity on infarct size, circulating cardiac biomarkers, myocardial mitochondrial respiratory capacity and mortality in adult Göttingen minipigs with experimentally induced MI and diet-induced obesity.

## Results

### Animals

**Fig. 1:**
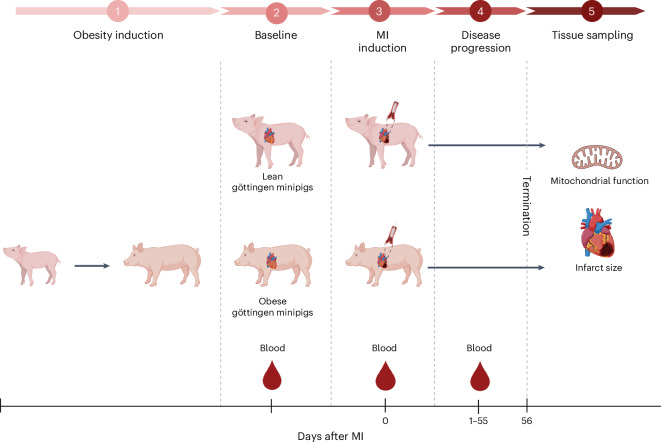
Study setup, including induction of MI in obese and lean Göttingen minipigs. Blood samples were collected during the study period, and cardiac tissue was collected for analyses at the end of the study. Created with BioRender (Debes, K. (2025); https://BioRender.com/y11e889).

The study setup is shown in Fig. 1. The obese animals were older than the lean animals at study start and had significantly higher body weight, both at study start and at study end (Table 1). Biochemical and metabolic parameters are summarized in Table 2. Total cholesterol, low-density lipoprotein (LDL) and high-density lipoprotein (HDL) were significantly higher in obese animals at study start as well as at termination. Hematological parameters are available in Supplementary Table 1.

**Table 1:** Details of study population and excluded animals

	Lean	Obese	*P* value
Initial study population	*n* = 18	*n* = 20	
Age (months)	12 [1–217]	19 [15–20]	**<0.001**
Body weight at baseline (kg)	25.0 [23.4–36.5]	60.0 [55.9–64.6]	**<0.001**
Coronary occlusion not obtained	6% (1/18)	15% (3/20)	0.606
Mortality	12% (2/17)	53% (9/17)	**0.025**
Dead during MI induction	12% (2/17)	47% (8/17)	0.057
Dead during trial period	0% (0/17)	6% (1/17)	1.000
Excluded owing to premature deflation of balloon/interrupted occlusion (risk of preconditioning)	0% (0/17)	6% (1/17)	1.000
Total number of excluded animals	17% (3/18)	65% (13/20)	**0.004**
Final study population	*n* = 15	*n* = 7	–
Body weight at termination (kg)	26.0 [24.0–29.1]	58.0 [56.9–65.4]	**<0.001**

Data (age and body weight) are presented as median and IQR (in square brackets). *P* values less than 0.05 are bold to indicate statistically significant differences between lean and obese groups (Fisher’s exact or Wilcoxon rank sum).

**Table 2:** Biochemistry measurements in plasma from lean and obese Göttingen minipigs at baseline and 8 weeks after MI

		Lean (*n* = 15)		Obese (*n* = 7)	
Parameter	Unit	Baseline	8 weeks post-MI	Baseline	8 weeks post-MI
Glucose	mmol/l	3.83 [3.67–4.14]	4.08 [3.65–4.81]*	4.30 [4.16–5.19]^#^	7.16 [4.64–8.25]*^#^
Lactate	mmol/l	1.14 [0.58–1.32]	0.91 [0.62–1.06]	0.95 [0.79–1.06]	1.52 [0.89–3.85]*^#^
Triglycerides	mmol/l	0.56 [0.44–0.69]	0.45 [0.39–0.59]	0.52 [0.39–0.66]	0.51 [0.44–0.58]
FFA	mmol/l	0.19 [0.08–0.28]	0.29 [0.07–0.49]	0.25 [0.09–0.30]	0.73 [0.29–0.93]^#^
Cholesterol	mmol/l	1.83 [1.59–2.03]	1.66 [1.53–1.97]	4.20 [3.36–5.33]^#^	3.95 [3.09–4.93]^#^
HDL	mmol/l	1.1 [1.0–1.2]	1.0 [0.9–1.3]	2.3 [1.7–2.7]^#^	2.0 [1.9–2.8]^#^
LDL	mmol/l	0.66 [0.58–0.82]	0.75 [0.54–0.86]	2.27 [1.70–2.96]^#^	2.11 [1.56–2.51]^#^
GGT	U/l	66 [50–135]	85 [67–128]	99 [77–222]	65 [58–255]
Albumin	g/l	46 [43–47]	50 [48–52]*	51 [49–54]^#^	50 [48–52]
Urea	mmol/l	1.7 [1.4–2.1]	1.8 [1.3–2.4]	1.9 [1.7–2.1]	1.7 [1.5–1.7]*
CK	U/l	282 [209–597]	276 [263–392]	283 [228–1071]	257 [215–489]

Data presented as median and IQR (in square brackets). *P < 0.05; Wilcoxon signed-rank test for paired samples at baseline and 8 weeks after MI. ^#^*P* < 0.05; Wilcoxon rank-sum test for significant differences between lean and obese groups. CK, creatine kinase; FFA, free fatty acids; GGT, gamma-glutamyl transpeptidase.

### Model success rate and causes for animal exclusion

Coronary occlusion was not achieved in one lean and three obese animals owing to tortuous left anterior descending coronary arteries (LADs) (Table 1). Mortality was significantly higher in obese compared with lean animals (9/17 obese (53%) versus 2/17 lean (12%); Fisher’s exact test, *P* = 0.025) and was mainly associated with intraprocedural refractory ventricular fibrillation (VF) (Table 1). One of the obese animals died of unknown causes on day 52.

In one obese surviving animal, premature deflation of the balloon was used as a final attempt to resuscitate the animals from VF, but the individual was subsequently excluded from the study population owing to the risk that balloon deflation could affect the infarct size (Table 1).

### Electrocardiographic changes and arrhythmias during coronary occlusion

**Fig. 2:**
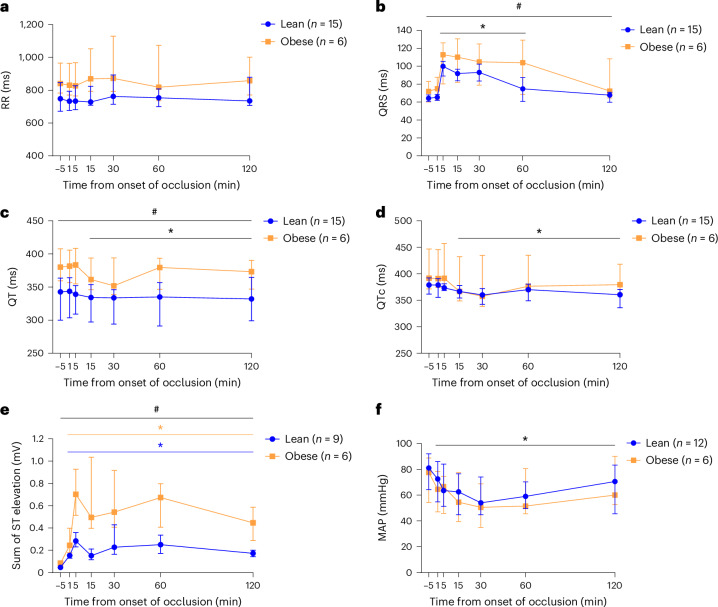
Electrocardiographic parameters and MAP recorded during coronary occlusion in lean and obese female Göttingen minipigs. **a**, RR interval. **b**, QRS duration. **c**, QT interval. **d**, QT interval corrected by Fredericia’s formula (QTc). **e**, Sum of ST elevation. **f**, MAP. ECG and blood pressure data are missing from one obese animal. In addition, ST elevation and MAP data are missing owing to technical issues in five and three lean animals, respectively. Data are presented as medians and IQR. Linear mixed-model analysis with the individual animal included as random effect. Significant differences between groups are indicated with a black hashtag. Significant differences from baseline values (5 min before occlusion) within individual groups are illustrated with colored asterisks, and when both groups are considered together with a black asterisk. ^#^*P* < 0.05; *P < 0.05. Data on ST elevation were log-transformed to obtain a better fit with the linear model.

There were no significant differences in the length of RR intervals reflecting heart rate between lean and obese animals, and heart rate remained stable during coronary occlusion (Fig. 2a). Obese animals had longer QRS duration compared with lean animals (linear mixed model, *P* = 0.0052), and the QRS duration was prolonged relative to baseline values from 5 to 60 min after onset of occlusion in both groups (linear mixed model, all *P* < 0.0001) (Fig. 2b). The unadjusted QT interval was also significantly longer in obese animals compared with the lean group (linear mixed model, *P* = 0.018), whereas this difference was not significant when the QT interval was corrected for heart rate (QTc) (linear mixed model, *P* = 0.059). In both groups, QT (linear mixed model, *P* ≤ 0.0007) and QTc (linear mixed model, *P* ≤ 0.0013) intervals were significantly shortened 15 min after onset of occlusion and throughout the occlusion period (Fig. 2c,d). ST-segment elevation developed in both groups during occlusion with more extensive ST elevation in the obese animals (linear mixed model, *P* ≤ 0.0050) (Fig. 2e). Finally, mean arterial pressure (MAP) showed no group differences but was significantly decreased compared with baseline during occlusion in both groups (linear mixed model, all *P* ≤ 0.0015) (Fig. 2f). Data on electrocardiography (ECG) and MAP values are provided in Supplementary Table 2.

**Fig. 3:**
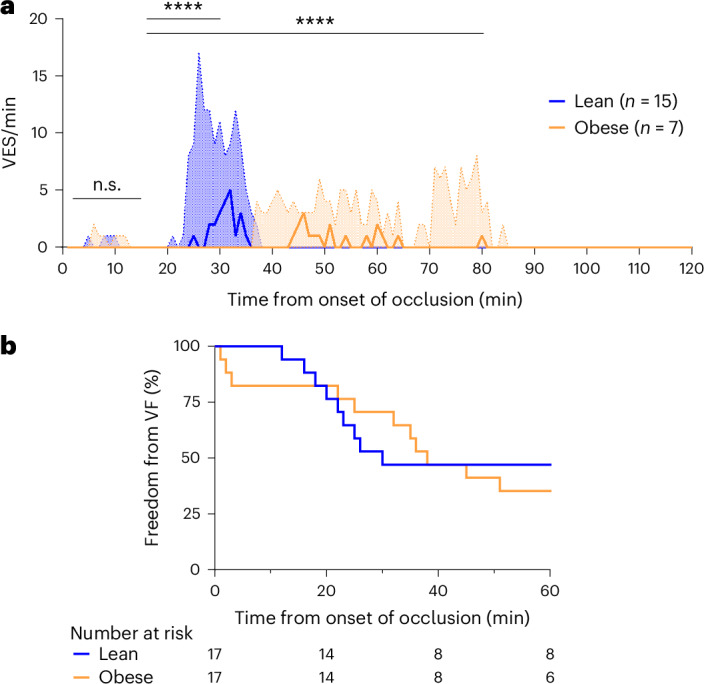
Arrhythmic activity during coronary occlusion in lean and obese female Göttingen minipigs. **a**, The number of VES counted per minute during 120-min coronary occlusion. Phase Ia occurred from 2 to 15 min after occlusion in both groups with no significant differences in timing or number of VES. Phase Ib occurred from 20 to 40 min in the lean group, whereas it was both delayed and extended in the obese group where it lasted from 35 to 80 min after occlusion. ****P < 0.0001 showing statistical significance in both permutation test and Wilcoxon rank-sum test comparing the number of VES in phase Ib (16–30 min) and phase Ib extended (16–80 min). **b**, Freedom from VF during the initial 60 min of occlusion. The time until onset of first VF was similar (nonsignificant, n.s.) in the lean and obese animals.

Ventricular extrasystoles (VES) appeared to be concentrated in two distinct phases (phases Ia and Ib) during occlusion as shown in previous publications^21,22^ but at different time periods in the two groups (Fig. 3a). Both timing (permutation test, *P* < 0.0001) and number of VES (permutation test, *P* < 0.0001) showed significant group differences, with lean animals having more VES early in the occlusion period, while obese animals showed more VES in the delayed phase. Eleven out of 17 (65%) obese animals and 9 out of 17 (53%) (Fisher’s exact test, *P* = 0.73) lean animals developed VF during coronary occlusion. Time from onset of occlusion until onset of first VF (Fig. 3b) and number of animals with more than one episode of VF were not different between groups. The median number of defibrillations per animal was 12 in the obese animals compared with 4 in the lean animals with intraprocedural VF. Furthermore, in 8 of the 11 (73%) obese animals with VF and in 1 of the 9 (11%) lean animals with VF (Fisher’s exact test, *P* = 0.010) defibrillation, chest compression and one to two doses of 1 mg intravenous (IV) adrenalin were additionally administered.

### Myocardial infarct size

**Fig. 4:**
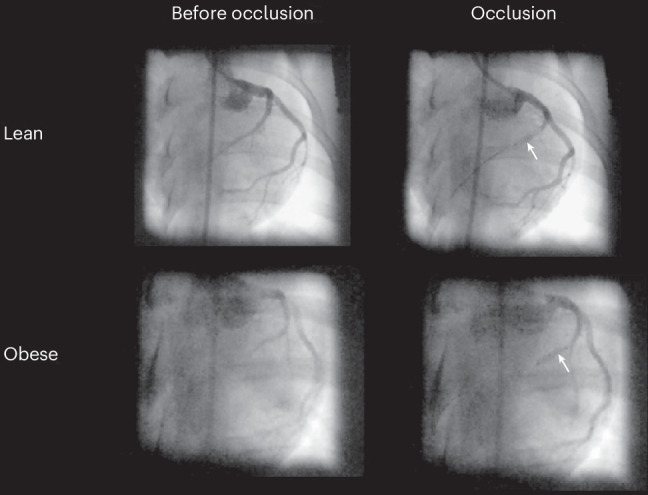
Angiographic images before and during coronary occlusion of the LAD in a lean and an obese female Göttingen minipig. The white arrows indicate balloon occlusion.

The occlusion was placed in the LAD distal to the first diagonal branch (D1) in 10/17 (59%) obese animals and in 13/17 (76%) lean animals. For the remaining animals, the position was distal to the second diagonal branch (D2). There was no significant difference in balloon position between groups (Fisher’s exact test, *P* = 0.46) (Fig. 4).

**Fig. 5:**
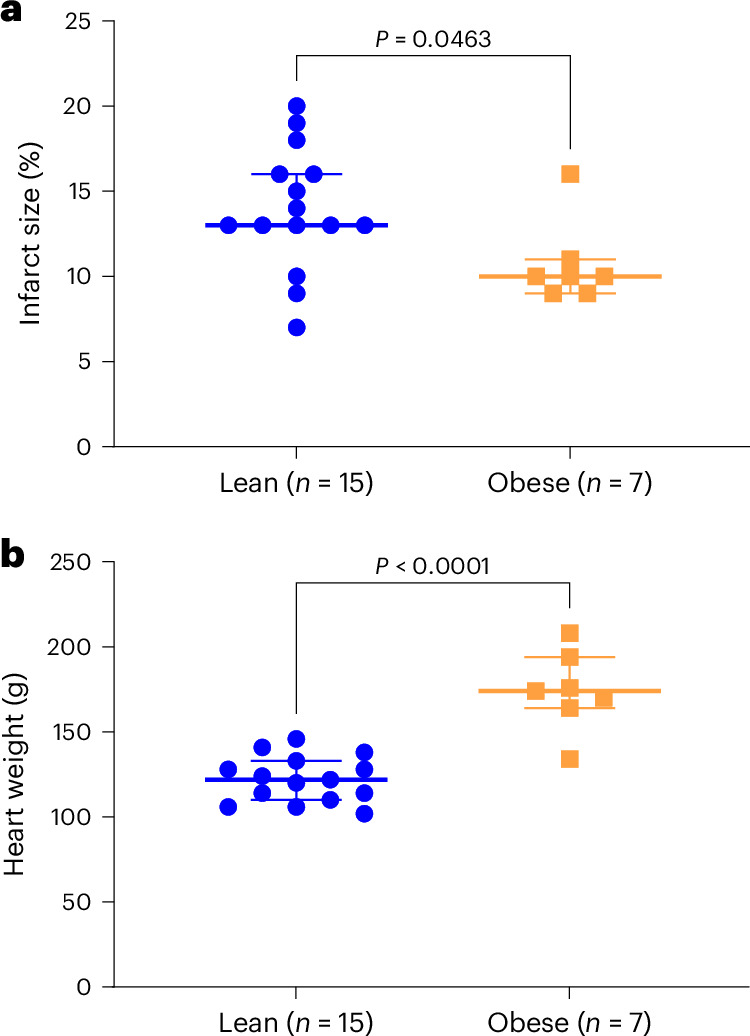
Gross pathology findings in lean and obese female Göttingen minipigs. **a**, Myocardial infarct size as a percentage of left ventricular cross-sectional area evaluated by TTC staining. **b**, Weight of the heart. Data presented as median and IQR. Wilcoxon rank-sum test.

Myocardial infarct size assessed ex vivo by staining with 2,3,5-triphenyltetrazolium chloride (TTC) 8 weeks after MI was significantly smaller in obese (median 10% [interquartile range (IQR) 9–11%]) compared with lean animals (13% [13–15%]) (Fig. 5a, Wilcoxon rank-sum test, *Z* = −2.00, *P* = 0.046). Heart weight was significantly increased in the obese animals (174 g [164–194 g]) compared with the lean animals (122 g [110–133 g]) (Fig. 5b, Wilcoxon rank-sum test, *Z* = 3.49, *P* < 0.0001).

### Circulating cardiac biomarkers

**Fig. 6:**
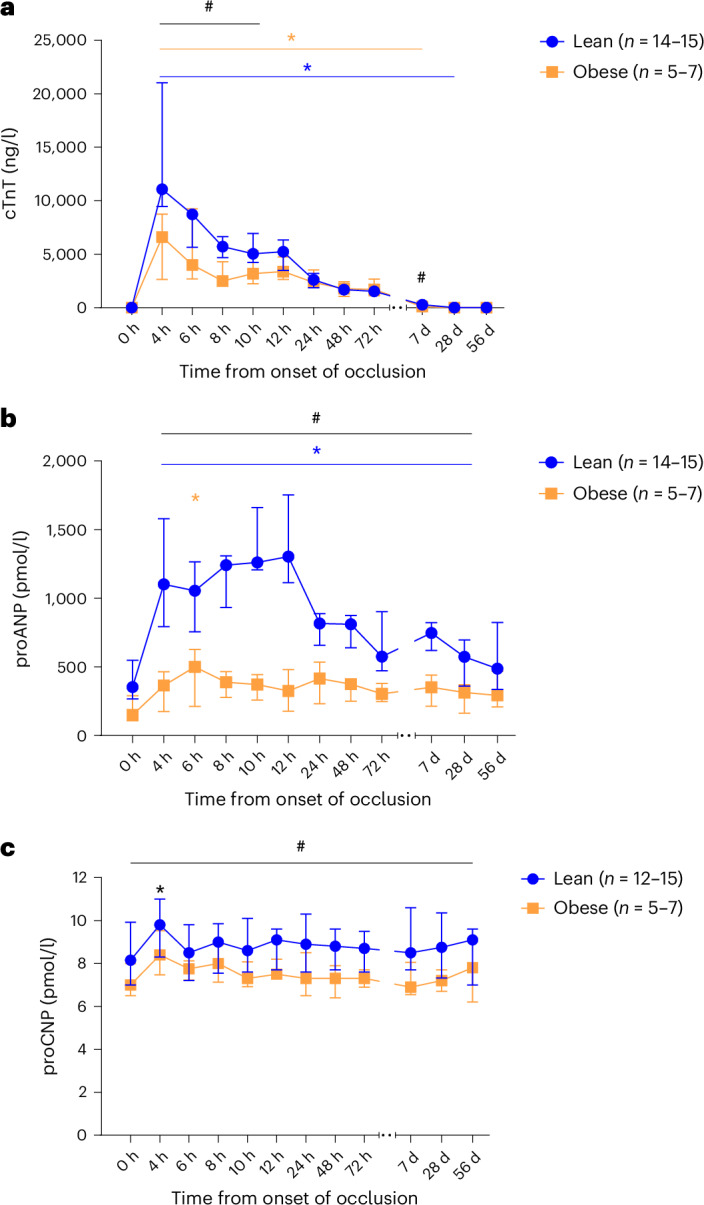
Plasma levels of circulating cardiac biomarkers after coronary occlusion in lean and obese female Göttingen minipigs. **a**, cTnT. **b**, proANP. **c**, proCNP. Data presented as median and IQR. Linear mixed-model analysis with the individual animal included as a random effect. Significance differences between groups are indicated with a black hashtag. Significant differences from baseline values before occlusion (hour 0) within individual groups are illustrated with colored asterisks, and when both groups are considered together with a black asterisk. ^#^*P* < 0.05; *P < 0.05. Data on cTnT and proANP were log-transformed to obtain a better fit with the linear models. Two outliers were identified in the statistical model of cTnT, but statistically significant and nonsignificant findings were unchanged if the two outliers were excluded. Missing samples are indicated in the graph legends.

cTnT showed a time-dependent group difference (linear mixed model, *P* = 0.0002). Significantly lower levels of cTnT were found in the obese compared with the lean animals from 4 to 10 h (linear mixed model, all *P* ≤ 0.040) and at day 7 (linear mixed model, *P* < 0.0007) after occlusion. Furthermore, the cTnT levels were elevated compared with baseline values until day 7 in the obese animals (linear mixed model, all *P* < 0.0001) and until day 28 in the lean animals (linear mixed model, all *P* ≤ 0.0014) (Fig. 6a).

The circulating concentration of pro-atrial natriuretic peptide (proANP) was also shown to be lower in the obese group compared with the lean group from 4 h after onset of occlusion to day 28 (linear mixed model, all *P* < 0.037), but not at baseline (*P* = 0.069) or day 56 (*P* = 0.14). Moreover, the lean group had increased proANP concentrations compared with baseline values at all time points (linear mixed model, all *P* < 0.016), but not day 56 (*P* = 0.075). The obese group had increased proANP concentration only 6 h after onset of occlusion compared with baseline level (linear mixed model, *P* = 0.020) (Fig. 6b).

Pro-C-type natriuretic peptide (proCNP) levels were likewise significantly lower in obese compared with lean animals (linear mixed model, *P* = 0.016), but proCNP levels were in both groups significantly increased from baseline levels only at 4 h after onset of occlusion (linear mixed model, *P* = 0.0016) (Fig. 6c). Data on biomarker levels are provided in Supplementary Table 3.

### Mitochondrial respiratory capacity

**Fig. 7:**
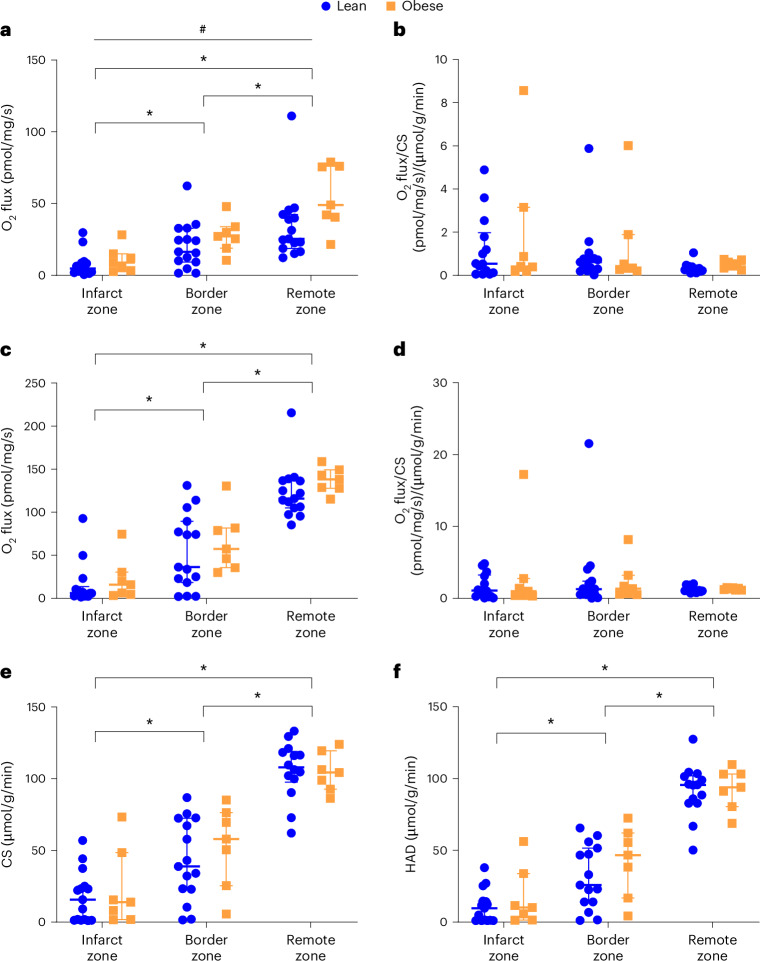
Mitochondrial function 8 weeks after MI in lean and obese female Göttingen minipigs. **a**, Complex-I-linked respiratory capacity. **b**, Intrinsic complex-I-linked respiratory capacity (complex I/CS ratio). **c**, Respiratory capacity linked to complexes I + II. **d**, Intrinsic respiratory capacity linked to complexes I + II (complex I + II/CS ratio). **e**, CS activity. **f**, HAD activity. Data presented as median and IQR. Linear mixed-model analysis with individual animals included as a random effect. Significance differences between groups are indicated with a black hashtag. Significant differences between zones when both groups are considered together are indicated with a black asterisk. ^#^*P* < 0.05; *P < 0.05. Log transformation was performed on O_2_ flux data from complex I to obtain a linear fit. *n* = 15 (lean) and 7 (obese).

Complex-I-linked mitochondrial respiratory capacity was overall significantly higher in obese animals (linear mixed model, *P* = 0.013) and showed significant differences between myocardial zones, with diminished activity in a stepwise manner, with lowest activity in the infarcted myocardium compared with the border zone and the remote zone (linear mixed model, all *P* ≤ 0.0060) (Fig. 7a). However, when complex-I-linked capacity was corrected for citrate synthase (CS) activity, as a surrogate for the number of mitochondria in the tissue sample, these differences dissipated (Fig. 7b). Respiratory capacity linked to complexes I + II did not differ between groups but was likewise diminished in the infarct and border zones in a stepwise manner (linear mixed model, all *P* ≤ 0.0001) (Fig. 7c); however, these differences were likewise leveled out by correction for CS activity (Fig. 7d). Both CS and 3‐hydroxyacyl‐CoA dehydrogenase (HAD) activity also showed significant stepwise differences between myocardial zones with decreased activity in the infarct and border zones but no differences between the lean and obese groups (linear mixed models, all *P* ≤ 0.0002) (Fig. 7e,f). Data on mitochondrial respiratory capacity are provided in Supplementary Table 4.

## Discussion

This study presents the impact of obesity in a model of MI in Göttingen minipigs. With few exceptions, MI was successfully induced across the lean and obese minipig phenotypes. Obese animals had significantly longer QRS and unadjusted QT intervals as well as more extensive ST-segment elevation during coronary occlusion compared with lean animals. Although malignant arrhythmias were similar in the two groups during occlusion, intraprocedural mortality was significantly higher in obese animals. However, when following up 8 weeks later, obese animals had lower levels of circulating cardiac biomarkers and smaller infarcts compared with lean animals. Finally, obese animals had significantly higher mitochondrial complex I respiratory capacity compared with lean animals. However, no difference between the groups was found when adjusting for CS activity reflecting the number of mitochondria in the tissue sample.

In general, obese animals showed a different response to the induction of MI compared with lean animals, and the induction procedure proved to be considerably more challenging in obese animals (Table 1). Despite a similar prevalence of VF in obese and lean animals during occlusion, intraprocedural mortality was about fourfold greater in obese animals compared with lean animals, mainly driven by VF. This finding has translational interest because MI-induced VF is reported to be the main cause of cardiac sudden death in human patients^23^. In the obese pigs, VF was refractory to defibrillation, IV adrenalin and chest compressions, whereas the lean animals were frequently cardioverted by a single defibrillation. This is in contrast to a study of defibrillation in human patients, which did not find an association between BMI and success rates^24^. Furthermore, the study investigated the effect of the energy level used for defibrillation, because the greater transthoracic impedance in patients with obesity may influence the energy delivered to the heart; however, an association was not found^24^. Even so, we used a higher energy level to defibrillate the obese animals (360 kJ versus 200 kJ in the lean animals). Another factor that can influence the success rate of defibrillation is the position of the defibrillation pads. Vector-change defibrillation, where the pads are changed from the standard anterior–lateral position to the anterior–posterior position, has been shown to be effective in refractory cases of VF in human patients^25^. As the anterior–posterior position was used, the explanation for the resistance to cardioversion in the obese animals must be found elsewhere. The added stress of the anesthesia from being obese (dorsal recumbency, compromised respiration and potential suboptimal dosing of anesthetics relative to lean body mass) may have an impact. However, ECG changes (prolonged QRS and more extensive ST elevation) during occlusion may also suggest an altered electrophysiology (Fig. 2). Ventricular ectopy also showed distinct patterns between the two groups (Fig. 3a). Phase Ib was both delayed and extended in obese animals compared with previous studies^21,22,26^, and the number of VES was higher than in the lean group.

Surviving obese animals had significantly smaller infarcts 8 weeks after MI (Fig. 5). A possible explanation could be that the obese animals in which the balloon occlusion rendered the largest myocardial infarcts died in the acute stage during MI induction and did not survive to the quantification of infarct size 8 weeks later. Some human studies have proposed that the obesity paradox also affects myocardial infarct size^8,27,28^. Sohn et al.^8^ found overweight to be an independent predictor of smaller infarct size based on cardiac magnetic resonance imaging (cMRI) in 193 patients, and likewise, Pingitore et al.^28^ showed smaller infarcts in patients with obesity measured by cMRI in a study of 89 patients with previous MI. These findings were contradicted in a more recent study by Reinstadler et al.^29^ who examined 426 patients with cMRI within 2 days after MI without finding any association between BMI and infarct size. Whether the infarct size was indeed smaller in all the obese animals of this study—and not just in the ones that survived the induction procedure—remains speculative. It could be speculated that high-fat diet may have induced preconditioning against cellular necrosis, for example, by upregulation of cardioprotective genes in the myocardium.

In agreement with final infarct size, we also observed lower levels of circulating cardiac biomarkers in obese animals (Fig. 6). Although there was no difference between groups at baseline, obese animals had significantly lower cTnT levels immediately after MI (Fig. 6a), which matched the significantly smaller infarcts 8 weeks later. The natriuretic peptide proANP likewise showed no group difference at baseline but was significantly lower in obese animals for 28 days after onset of occlusion (Fig. 6b). This finding agrees with human studies showing impaired response of natriuretic peptides in individuals with obesity^12^. A novel finding of this study was the significantly lower levels of proCNP found in obese animals throughout the study period, even at baseline (Fig. 6c). This finding is interesting as higher concentrations of proCNP have recently been shown to be an independent risk marker for mortality in female patients with MI^30^.

Cardiac metabolism assessed by mitochondrial respiratory capacity was only mildly affected by obesity in the present study. Complex-I-linked respiratory capacity seemed to be higher in obese animals compared with lean animals (Fig. 7a). However, this result may merely reflect different numbers of mitochondria in the tissue samples, as there was no difference between groups when the O_2_ flux was corrected for CS activity (Fig. 7b). In agreement, Christiansen et al.^31^ studied the respiratory capacity in Göttingen minipigs without MI and found no difference between lean and obese animals. Instead, the differences observed in mitochondrial respiratory capacity in this study were related to the different myocardial tissue sampling zones, with decreased respiratory capacity in a stepwise manner and lower levels in infarct and border zones compared with the remote zone. Myocardial ischemia can lead to mitochondrial changes, including decreased respiratory capacity, CS activity and increased production of reactive oxygen species^32–36^. However, given the different levels of scar tissue formation in the different zones, a possible explanation could also be that tissue with active mitochondria may have been displaced by fibrosis.

The study has limitations. Porcine cardiovascular models have limitations despite being described as superior to rodent models^37^. Göttingen minipigs develop diet-induced obesity and coronary atherosclerosis^38^, but obesity seems not to be associated with systemic hypertension in this pig breed^39^; in addition, studies on endothelial dysfunction are limited^37^. A recent study stated that the Ossabaw pig breed may be a better model for human cardiovascular diseases than Göttingen minipigs owing to their genetic background and development of a fuller human-like obesity-related metabolic syndrome profile^40^. However, controlled studies including both pig breeds where age, sex and diet compositions are taken into account are missing^37^. Furthermore, it is important to be aware of differences between porcine MI models and settings in human patients with MI. For example, antiarrhythmic agents are often used in porcine MI models but are uncommon in patients at the onset of an MI.

In the present porcine MI model, blood reflow after balloon deflation was not quantified and a ‘no-reflow’ phenomenon was, thus, not detected or quantified. Rare no-reflow is associated with poor recovery of ventricular function, and no-reflow has been reported as an independent predictor of poor outcome after MI in humans^41^. Evans blue staining estimating myocardial area at risk was not included as a part of the infarct size quantification despite this method being considered as the gold standard in experimental studies^42^. Infarct size was quantified only using TTC myocardial staining differentiating viable and necrotic myocardial tissue. Furthermore, myocardial gene expression analyses were not performed. It could have been interesting to study whether cardioprotective genes were induced in obese pigs.

A limitation of the respirometric results is that the analyses were performed in vitro, which may not reflect mitochondrial function in vivo. Moreover, temporality of the mitochondrial function measurement was not included in the present study, but it is relevant for future studies because good agreement between mitochondrial function measurements in permeabilized myocardial biopsies and ischemia–reperfusion damage has been reported in human patients as well as in Göttingen minipigs^43^. An additional study limitation was that dobutamine was administered during the stress test in the hours preceding euthanasia and tissue sampling. Dobutamine has previously been shown to improve mitochondrial function in the infarcted myocardium^44^. Hence, the mitochondrial respiratory capacity measured in this study may be relatively higher than what could be observed in the untreated myocardium. However, as both the obese and lean animals underwent the dobutamine stress test, this is not expected to obscure a potential group difference.

Another limitation of this study is the lack of age-matched groups. The obese animals were available from other in-house studies where they had been fed western-style diet for up to 6 months to attain their obese state and were older than the lean animals. However, in both groups the animals had reached sexual maturity and adult size, although not fully grown. Previous studies have reported that mitochondrial function decreases with advancing age^45^. However, the age difference between the two groups was relatively limited.

Finally, we used a closed-chest model of infarction to mimic the human scenario with coronary occlusion and primary angioplasty. The closed-chest model also aided the early awakening of animals without the surgical trauma due to sternotomy. Defibrillation may, however, be less effective due to the large thorax of the pig. The MI outcomes could, therefore, be different in an open-chest model. In conclusion, induction of MI is feasible in obese Göttingen minipigs. However, obese animals had higher intraprocedural mortality from refractory VF than lean control animals. Based on the ECG findings (longer QRS, QT, higher ST elevation and altered pattern of ventricular arrhythmias), this may reflect an altered electrophysiology in the obese minipig heart.

Paradoxically, the obese animals that survived the procedure had smaller infarcts and showed lower response in circulating cardiac biomarkers. However, it is important to be aware that the obese pigs that showed smaller infarct size and lower circulating levels of cardiac markers compared with the lean pigs were a survivor subset of the obese pig population. Only minor differences in cardiac mitochondrial respiratory capacity were detected between lean and obese animals.

## Methods

### Animals

Eighteen lean and 20 obese female Göttingen minipigs were recruited for the study (Ellegaard Göttingen minipigs A/S). Some of the animals were a part of other publications focusing on other endpoints^46–48^. These publications include parts of the baseline characteristics. The obese animals had also been included in other in-house studies testing unrelated pharmacological substances but had been through a relevant washout period before included in the current study. Sample size power calculation was not performed due to the explorative design of the study. Animals were not randomized into groups, and it was not possible to blind in vivo procedures owing to the size of the pig. All animals had free access to water and bedding material and were single-housed due to implanted IV catheters. Lean animals were fed a standard minipig diet (Altromin 9033, Brogaarden Aps) twice daily according to body weight. The obese animals were fed a western-style diet (25% fat, 23% fructose and 0.5% cholesterol) (custom made, Foulum, Aarhus University, Aarhus, Denmark)^49^. The western-style diet feeding regimen was continued for up to approximately 6 months (156 days [24–162 days] before induction) to induce obesity and was restricted, if needed, to maintain stable weight during the trial period. All animals were closely monitored for mobility, behavior and well-being throughout the study, and humane endpoints included dyspnea and other signs of circulatory distress. The study was approved by the local Ethical Research Council and the Danish Animal Inspectorate (license number 2018-15-0201-01414) in compliance with European legislation.

All included animals underwent MI induction by balloon occlusion of the LAD with continuous three-lead ECG and MAP recording, and blood sampling for circulating cardiovascular biomarkers. The procedures were performed in lean and obese animals in interchanging batches. Eight weeks later, the final study population was subjected to a terminal procedure including blood sampling and in vivo assessment of the cardiac functional reserve during a dobutamine stress test (data not shown). Directly after this assessment, the animals were euthanized for post-mortem in vitro analyses of myocardial infarct size and mitochondrial respiratory capacity. The study protocol was prepared before the study, and all in vitro analyses were performed blinded to group identity.

### Venous access and blood sampling

The majority of the obese pigs were equipped with one semipermanent auricular catheter (BD Careflow 3Fr 200 mm, Argon Medical) and a central venous catheter (Cook C-TPNS-6.5-90-REDO, William Cook Europe ApS) from a previous study^48^. Five to 7 days before MI induction, the lean pigs as well as obese pigs without catheters or with dysfunctional auricular catheters had catheters placed using the Seldinger technique as previously described^50^. Animals received an IV infusion of ampicillin (1.0 ml per 10 kg body weight) (Pentrexyl, Corden Pharma Latina S.p.A.) and sterile saline (1 g solved in 10 ml sterile saline). They were treated with per oral (PO) meloxicam (Inflacam 15 mg/ml, Virbac) for 4 days postoperatively. Maintenance of catheters was achieved by flushing them twice weekly throughout the trial period.

### Anesthesia and analgesia

Animals were premedicated with 0.02 mg/kg meloxicam by intramuscular (IM) injection (Metacam 20 mg/ml, Boehringer Ingelheim) and 15 mg/kg amoxicillin IM (Noramox prolongatum 150 mg/ml, Boehringer Ingelheim). Anesthesia was induced by a mixture of 125 mg zolazepam + 125 mg tiletamine (Zoletil 50, Virbac) diluted by 125 mg ketamine (Ketaminol 100 mg/ml, MSD Animal Health) + 130 mg xylazine (Xysol Vet. 20 ml/mg, ScanVet) + 25 mg butorphanol (Morphasol Vet. 10 mg/ml, AniMedica) to a total volume of 10.25 ml and injected intramuscularly at a dose of 1.0 ml mixture/10 kg body weight. Anesthesia was maintained by continuous infusion of fentanyl 5 µg/kg/h (Fentanyl-Hameln 50 mg/ml, Hameln) and propofol (Propolipid 10 mg/ml, Fresenius Kabi AB). Propofol was administered using stepwise downtitration with dosages for lean animals being 15 mg/kg/h before occlusion, 10 mg/kg/h during occlusion and 5 mg/kg/h after balloon deflation. The propofol dosages for obese animals were reduced to adjust for lean body mass and facilitate faster recovery^51^: 12.5 mg/kg/min before occlusion, 7.5 mg/kg/min during occlusion and 5 mg/kg/min after balloon deflation. Postoperative analgesia consisted of 0.08 mg/kg buprenorphine IM (Vetergesic 0.03 mg/ml, Ceva), buprenorphine transcutaneous patches 3.5 µg/kg/h for 72 h (Transtec, Mundipharma) and 0.4 mg/kg meloxicam PO for 4 days (Inflacam 15 mg/ml, Virbac). In addition, animals received 15 mg/kg amoxicillin PO for 3 days (Paracillin Vet 800 mg/g, MSD Animal Health).

### Electrophysiology and blood pressure

All surgical procedures were performed with continuous monitoring of ECG, capnography, pulse oximetry and temperature (Mindray). Furthermore, ECG data from three orthogonal leads (Frank configuration) were recorded on a Quad Bio Amp electrocardiograph switch box with a power lab and LabChart (AD Instruments). The following ECG parameters were measured as previously described^52^: RR, QT and QT corrected for heart rate (QTc = (QT/RR)^1,3^) intervals, QRS duration and sum of ST elevation 60 ms after the J point.

Invasive arterial blood pressure was recorded using a pressure-tip catheter (Millar) accessing the abdominal aorta through a 6 F sheath (Avanti+, Cordis) in the left femoral artery.

### Induction of MI

The right femoral artery was accessed percutaneously using a 6 F sheath (Avanti+, Cordis) and Seldinger’s technique. Once all arterial sheaths were in place, animals received 5,000 IU heparin IV (Heparin LEO 5,000 IU/ml, Leo Pharma) as an anticoagulant. An H-stick 6 F guiding catheter (Vista brite tip, Cordis) was advanced under fluoroscopic guidance to the left coronary inlet, and an angiogram was performed using iodinated contrast (Visipaque, GE Healthcare). A percutaneous transluminal coronary angioplasty balloon catheter (Ikazuchi Zero, Cardinal Health) was advanced over a 0.014′ diagnostic guidewire (Stabilizer, Cordis) into the LAD. The balloon (diameter 2.00–2.75 mm × 15 mm in length) was inflated distal to the first (D1) or second (D2) diagonal branch corresponding to the proximal LAD on the basis of individual anatomy. After 120 min (ref. ^19^), the balloon was deflated, allowing reperfusion of the ischemic myocardium.

Arrhythmia prevention consisted of 10 mg/kg amiodarone (Cordarone, Sanofi) diluted in saline administered intravenously over a 20-min period before occlusion. This was supplemented by boluses of 2 mg/kg lidocaine IV (Lidokain Mylan 20 mg/ml) at approximately 5 min and 25 min after occlusion. In the event of VF, a preattached cardiac defibrillator (Lifepak20, Medtronic) was employed using 200 kJ for lean animals and 360 kJ for obese animals. Defibrillation pads were placed in anterior–posterior position^25^. When defibrillation alone was not enough to achieve cardioversion, 1 mg adrenalin IV (Adrenalin DAK 1 mg/ml, Takeda Pharma) and 2 mg/kg lidocaine IV were administered, and subcostal chest compressions were applied. After the MI procedure, arterial sheaths were removed, and hemostasis of both femoral arteries was achieved through 10 min of compression. While recovering from anesthesia, the animal was monitored using a portable oximetry device and received oxygen supplementation. In addition, the loin area was observed for any signs of hematoma.

All animals were treated with 7 mg/kg acetylsalicylic acid PO (Hjertemagnyl 75 mg, Takeda GmbH Oranienburg) for 28 days after MI to reduce the risk of thrombus formation.

### Blood sampling and analyses

Blood samples for hematology and biochemistry were collected at baseline and in the eighth week of the study through permanent venous ear catheters in awake animals in the morning before feeding. Hematological parameters were analyzed in ethylenediaminetetraacetic acid (EDTA)-stabilized whole blood within 3 h of collection (Sysmex XT-2000iv, Sysmex Nordic ApS). Blood samples for biochemistry were stabilized in EDTA, centrifuged (3,000*g*, 10 min, 4 °C) and frozen at −80 °C until analysis (Cobas 6000, Roche).

Additional plasma samples were collected for analysis of circulating cardiac biomarkers at baseline, 4, 6, 8, 10, 12, 24, 48 and 72 h, and 7 and 28 days after LAD occlusion and in the eighth week of the study. These were likewise EDTA stabilized, centrifuged and frozen at −80 °C until analysis. Circulating plasma levels of cTnT were measured in duplicate by Elecsys Troponin T hs assay (Cobas, Roche) with a lower detection limit of 5 ng/l. Plasma concentrations of the proANP and proCNP, precursors to ANP and CNP, respectively, were also quantified in duplicate by a processing-independent assay that measures total sum of forms translated and secreted as previously reported^53,54^.

### Myocardial tissue samples

Animals were euthanized by cardiac excision under general anesthesia directly after the terminal procedure. The heart was quickly explanted from thorax, rinsed in saline, dabbed dry and weighed. The ventricles were cut into 1-cm-thick slices starting at the apex and ending at the level of the mitral annulus.

Transmural myocardial tissue samples 4 mm in diameter were sampled from the infarct zone and border zone on the anterior left ventricular wall, and from a remote zone at the posterior aspect of the left ventricular for mitochondrial respiratory capacity analyses (Fig. 1). Myocardial tissue sampling was guided by the macroscopic appearance of the fibrotic changes. The border-zone sample was taken precisely at the demarcation between the fibrotic, infarcted tissue and the apparently healthy tissue. The tissue samples were collected from the middle of slice to avoid the cutting surface used for infarct size determination. Each individual tissue sample was divided into two pieces cut in half along the transmural axis. One sample was used for high-resolution respirometry and the other sample for analyses of CS and HAD activities.

The tissue samples for respirometry were kept in iced BIOPS preservation buffer as previously described^31^ and analyzed within 24 h of sample collection. The samples for CS and HAD analysis were snap frozen in liquid nitrogen and stored at −80 °C until analysis.

### Mitochondrial respiratory capacity analyses

Mitochondrial respiratory capacity in complexes I and I + II was assessed as described previously^31,55,56^. In brief, the myocardial tissue samples were dissected, permeabilized with 50 μg/ml of saponin and rinsed in MiR05 buffer^31^. Maximal mitochondrial respiratory capacity was measured in duplicate with high-resolution respirometry (Oxygraph O2k, Oroboros). Substrates to stimulate and inhibit the electron transport chain were added in this order: malate (2 mM) and pyruvate (5 mM) to evaluate state 2 respiration (leak), ADP (5 mM) and magnesium (3 mM) to evaluate state 3 respiration, glutamate (10 mM) to measure complex I respiration, cytochrome C (10 μM) to evaluate the integrity of outer mitochondrial membrane, succinate (10 mM) to measure complex I + II respiration and antimysin A (2.5 µM) to inhibit complex III activity.

HAD is an enzyme in the fatty acid β-oxidation and is used to assess the β-oxidation capacity^57^. CS can be measured as a marker of mitochondrial content in skeletal muscle^58^. CS and HAD activity were measured on Cobas 6000 (C 501, Roche Diagnostics) as described previously^31,59^. In brief, the tissue was homogenized (Tissuelyzer, Qiagen, Venlo) in a buffer (1,000 μl of 0.3 M K_2_HPO_4_ and 0.05% bovine serum albumin, pH 7.7, 2 min), 10% Triton was added reaching a final concentration of 0.1% Triton and the sample was diluted before measurement^31,59^. Respiratory capacity linked to complex I and complexes I + II normalized to CS were reported as respiratory capacity linked to intrinsic complex I and complexes I + II, respectively.

### Myocardial infarct size

After collection of tissue samples for mitochondrial analyses, the 1-cm-thick myocardial slices were stained using a 1% (w/v) 2,3,5-TTC (≥98% (HPLC), Sigma-Aldrich) and phosphate-buffered saline (Dulbecco’s phosphate-buffered saline, Sigma-Aldrich) solution, and the cross-sectional surfaces were stored as digitally scanned images. The myocardial infarct size was estimated by tracing the area of infarcted myocardium relative to the left ventricular area^60^ using the image analysis program ImageJ (National Institutes of Health).

### Statistical analysis

Data are presented as median and IQR. Group comparisons of lean and obese animals were made by Fisher’s exact test or Wilcoxon rank-sum test (Mann–Whitney *U* test). Paired samples within groups were analyzed using the Wilcoxon signed-rank test. A linear mixed model with the individual animal included as a random effect was used to assess the effect of group (lean versus obese) on parameters with repeated measures, that is, ECG parameters, blood pressure, circulating biomarkers and mitochondrial analysis measurements from different myocardial zones. Interaction between group and time as well as the individual effects of group, time and myocardial sampling zone were assessed. Residual homogeneity and normality were used to test model fit, and if the data did not fit, logarithmic transformation of data was performed. All statistical tests were performed in SAS Enterprise Guide except the analysis of differences between groups in timing of VES, which was assessed by permutation test in R (ref. ^52^).

### Reporting summary

Further information on research design is available in the Nature Portfolio Reporting Summary linked to this article.

## Online content

Any methods, additional references, Nature Portfolio reporting summaries, source data, extended data, supplementary information, acknowledgements, peer review information; details of author contributions and competing interests; and statements of data and code availability are available at 10.1038/s41684-025-01533-4.

## Supplementary information

Supplementary Information Supplementary Tables 1–4.

Reporting Summary

## Data Availability

The datasets used and analyzed during the present study are available from the corresponding author on reasonable request.
