# Supplementary material for: Impact of obesity on infarct size, circulating biomarkers, mitochondrial function and mortality in a Göttingen minipig myocardial infarct model
Source: Lab Anim (NY). 2025 Mar 27;54(4):103–11. doi: 10.1038/s41684-025-01533-4 (PMC11957994; doi:10.1038/s41684-025-01533-4)
Supplement: Supplementary file 1 — Supplementary Tables 1–4. [file 41684_2025_1533_MOESM1_ESM.pdf]

lab animal

Supplementary information

https://doi.org/10.1038/s41684-025-01533-4

Impact of obesity on infarct size, circulating biomarkers, mitochondrial function and mortality in a Göttingen minipig myocardial infarct model

In the format provided by the authors and unedited

Supplementary information

Supplementary Table 1 Haematology in lean and obese minipigs at baseline and 8 weeks post-MI.

	Unit	Lean (n=15)		Obese (n=5)	
		Baseline	8 weeks post-MI	Baseline	8 weeks post-MI
Haematology					
WBC	10^9/L	9.8 [8.3 - 12.4]	7.7 [7.3 - 10.9] *	8.8 [8.3 - 9.1]	10.4 [9.3 - 10.6]
Neutrophils	10^9/L	3.5 [2.5 - 4.1] (n=13)	2.6 [2.0 - 4.4]	1.6 [1.4 - 3.3]	3.4 [3.3 - 4.0]
Lymphocytes	10^9/L	5.3 [5.1 - 6.3] (n=13)	4.6 [3.8 - 5.6]	5.4 [5.0 - 6.0]	5.8 [5.2 - 6.3] #§
Monocytes	10^9/L	0.90 [0.62 - 1.06] (n=14)	0.54 [0.36 - 0.71] *	0.48 [0.45 - 0.57] #	0.52 [0,51 - 0.76]
Eosinophils	10^9/L	0.36 [0.24 - 0.49] (n=14)	0.31 [0.23 - 0.44]	0.30 [0.26 - 0.31]	0.29 [0.25 - 0.30]
Basophils	10^9/L	0.03 [0.01 - 0.04]	0.02 [0.01 - 0.04]	0.02 [0.01 - 0.02]	0.01 [0.01 - 0.04]
RBC	10^9/L	6.9 [6.2 - 7.8]	6.3 [6.1 - 7.3]	7.3 [6.8 - 7.8]	7.2 [7.1 - 7.6]
Haemoglobin	mmol/L	8.4 [7.0 - 8.8]	7.9 [7.4 - 8.6]	7.8 [7.4 - 8.2]	7.8 [7.6 - 7.9] (n=4)
Haematocrit	L/L	0.40 [0.33 - 0.42]	0.38 [0.34 - 0.39]	0.38 [0.35 - 0.38]	0.37 [0.35 - 0.37] (n=4)
Platelets	10^9/L	436 [391 - 582]	457 [401 - 533]	401 [379 - 442]	392 [299 - 432]
Reticulocytes	10^9/L	35.7 [31.2 - 50.9]	32.4 [21.6 - 38.7]	43.4 [27.8 - 47.1]	39.3 [27.5 - 41.2]

Data presented as median and interquartile range [IQR]. * $P < .05$; Wilcoxon signed rank test for paired samples at baseline and 8 weeks post-MI. # $P < .05$; Wilcoxon rank-sum test for significant difference between lean and obese groups. §; significance driven by one high value. Reduced n due to missing values and/or analysis error is shown in parentheses. WBC; white blood cell count. RBC; red blood cell count.

Supplementary Table 2 Electrocardiographic parameters and mean arterial pressure (MAP) during occlusion

		**Time from onset of occlusion (min)**							**P-value for group difference**
	**Group**	**-5**	**1**	**5**	**15**	**30**	**60**	**120**	
RR (ms)	Obese	841 [783 - 966]	831 [771 - 964]	827 [765 - 968]	870 [793 - 1052]	872 [793 - 1130]	819 [736 - 1074]	858 [771 - 1002]	ns
	Lean	749 [672- 847]	733 [678 - 794]	734 [682 -827]	728 [708 - 824]	762 [715 - 893]	754 [700- 808]	735 [707 - 879]	
QRS (ms)	Obese	72 [67 - 80] #	75 [69 - 81] #	113 [87 - 122] *#	110 [86 - 128] *#	105 [80 - 123] *#	104 [77 - 123] *#	72 [67 - 89] #	.0052
	Lean	64 [61 - 68] #	65 [62 - 69] #	100 [89 - 105] *#	92 [84 - 97] *#	93 [84 - 102] *#	75 [61 - 87] *#	68 [60 - 71] #	
QT (ms)	Obese	380 [359 - 408] #	382 [357 - 406] #	383 [355 - 408] #	361 [346 - 394] *#	352 [339 - 394] *#	380 [347 - 394] *#	373 [347 - 390] *#	.0018
	Lean	343 [300 - 364] #	344 [304 - 364] #	339 [309 - 352] #	334 [297 - 354] *#	334 [294 - 346] *#	335 [291 - 357] *#	332 [299 - 365] *#	
QTc (ms)	Obese	392 [371 - 447]	391 [374 - 446]	392 [374 - 457]	367 [349 - 432] *	358 [338 - 435] *	376 [367 - 435] *	379 [363 - 418] *	ns
	Lean	379 [362 - 392]	379 [356 - 391]	374 [368 - 381]	367 [354 - 378] *	360 [342 - 372] *	370 [349 - 380] *	360 [336 - 370] *	
Sum ST (mV)	Obese	0.08 [0.08 – 0.11]#	0.24 [0.18 – 0.40]*#	0.70 [0.51 – 0.93]*#	0.50 [0.40 – 1.04]*#	0.54 [0.41 - 0.92] *#	0.67 [0.41 – 0.80]*#	0.45 [0.29 – 0.59]*#	.042 §
	Lean	0.05 [0.04 – 0.06]#	0.15 [0.13 – 0.17]*#	0.28 [0.23 - 0.36] *#	0.15 [0.12 – 0.21]*#	0.23 [0.16 – 0.43]*#	0.25 [0.17 – 0.34]*#	0.17 [0.14 – 0.20]*#	
MAP (mmHg)	Obese	78 [54 - 89]	65 [47 - 78] *	67 [46 - 75] *	55 [40 - 78] *	51 [35 - 69] *	52 [46 - 81] *	60 [53 - 90] *	ns
	Lean	81 [64 - 91]	73 [55 - 86] *	64 [51 - 84] *	63 [45 - 77] *	54 [45 - 74] *	59 [50 - 70] *	71 [46 - 83] *	

Data presented as median and interquartile range [IQR]. * $P < .05$; indicates significant difference from baseline values before occlusion (-5 min). # $P < .05$; indicates significant group difference between lean and obese animals at the given time point. §; indicates that the group difference is time dependent (i.e. significant interaction between group and time variables in the linear mixed model).

Supplementary Table 3 Plasma levels of circulating cardiac biomarkers after coronary occlusion

		Time from onset of occlusion (hours)					
**Biomarker (unit)**	**Group**	**0**	**4**	**6**	**8**	**10**	**12**
cTnT (ng/l)	Obese	5 [5 - 5] [a]	6604 [2630 - 8748] *#	3994 [2689 - 9231] *#	2493 [2100 - 4303] *#	3188 [2236 - 5343] *#	3384 [2646 - 4741] *
	Lean	5 [5 - 5] [a]	11076 [9474 - 21026] *#	8725 [5631 - 8985] *#	5713 [4680 - 6640] *#	5029 [4226 - 6946] *#	5238 [3486 - 6325] *
proANP (pmol/l)	Obese	151 [109 - 291]	365 [175 - 466] #	501 [213 - 627] *#	389 [278 - 466] #	372 [259 - 444]	325 [177 - 481] #
	Lean	353 [286 - 549]	1101 [794 - 1579] *#	1055 [756 - 1265] *#	1241 [934 - 1309] *#	1261 [1207 - 1660] *#	1304 [1115 - 1752] *#
proCNP (pmol/l)	Obese	7.0 [6.5 – 8.0] #	8.4 [7.5 – 9.5] *#	7.8 [7.2 – 8.1] #	8.0 [7.1 – 8.9] #	7.3 [6.9 – 8.1] #	7.5 [7.3 – 8.2] #
	Lean	8.2 [7.0 – 9.9] #	9.8 [8.3 – 11.0] *#	8.5 [7.2 – 9.8] #	9.0 [7.6 – 9.9] #	8.6 [7.6 – 10.1] #	9.1 [7.7 – 9.6] #

**Continued.**		**Time from onset of occlusion (hours)**						**P-value for group difference**
**Biomarker (unit)**	**Group**	**24**	**48**	**72**	**7 days**	**28 days**	**56 days**	
cTnT (ng/l)	Obese	2347 [2318 - 3519] *	1773 [1051 - 2425] *	1709 [1129 - 2666] *	91 [42 - 397] *	5 [5 - 10]	5 [5 - 5] [a]	.0002 §
	Lean	2598 [1864 - 3205] *	1691 [1391 - 1983] *	1532 [1401 - 1782] *	268 [152 - 377] *	6 [5 - 9] *	5 [5 - 5] [a]	
proANP (pmol/l)	Obese	416 [232 - 535] #	375 [251 - 388] #	304 [248 - 380] #	352 [215 - 442] #	314 [163 - 370] #	293 [210 - 468]	<.0001 §
	Lean	817 [659 - 888]*#	811 [640 - 875] *#	575 [472 - 902] *#	747 [620 - 822] *#	574 [361 - 697] *#	487 [336 - 824]	
proCNP (pmol/l)	Obese	7.3 [6.5 – 8.5] #	7.3 [6.4 – 7.9] #	7.3 [6.9 – 7.7] #	6.9 [6.6 – 8.1] #	7.2 [6.7 – 7.7] #	7.8 [6.2 – 7.9] #	.016
	Lean	8.9 [7.6 – 10.3] #	8.8 [7.7 – 9.6] #	8.7 [7.6 – 9.5] #	8.5 [7.7 – 10.6] #	8.8 [7.3 – 10.4] #	9.1 [7.0 – 9.6] #	

Data presented as median and interquartile range [IQR]. [a]; All except 1-2 values were below the detection limit of the troponin assay which was 5 ng/l. *P < .05; indicates significant difference from baseline values before occlusion (-5 min). # P < .05; indicates significant group difference between lean and obese animals at the given time point. §; indicates that the group difference is time dependent (i.e. significant interaction between group and time variables in the linear mixed model).

Supplementary Table 4 Mitochondrial respirometry data from three myocardial tissue sampling zones

		Tissue sampling zone			P-value for group difference
	**Group**	**Infarct zone (IZ)**	**Border zone (BZ)**	**Remote zone (RZ)**	
Complex I (pmol/mg/s)	Obese	9 [3 – 15] *[BZ, RZ] #	27 [19 - 34] *[IZ, RZ] #	49 [41 - 76] *[IZ, BZ] #	.013
	Lean	5 [2 – 9] *[BZ, RZ] #	16 [9 - 33] *[IZ, RZ] #	25 [19 - 42] *[IZ, BZ] #	
Complex I:CS ratio (pmol/mg/s)/(µmol/g/min)	Obese	0.4 [0.2 – 3.2]	0.3 [0.3 – 1.9]	0.5 [0.3 – 0.7]	ns
	Lean	0.5 [0.1 – 2.0]	0.6 [0.3 – 0.8]	0.3 [0.2 – 0.4]	
Complex I+II (pmol/mg/s)	Obese	16 [5 - 30] *[BZ, RZ]	57 [36 - 82] *[IZ, RZ]	138[128 - 149] *[IZ, BZ]	ns
	Lean	6 [3 - 14] *[BZ, RZ]	36 [18 - 89] *[IZ, RZ]	116 [105 - 137] *[IZ, BZ]	
Complex I+II:CS ratio (pmol/mg/s)/(µmol/g/min)	Obese	1.0 [0.4 – 2.8]	1.4 [0.6 – 3.2]	1.3 [1.2 – 1.5]	ns
	Lean	1.1 0.3 – 3.3]	1.3 [0.5 – 2.4]	1.1 [0.9 – 1.4]	
Citrate synthase (µmol/g/min)	Obese	14 [2 - 48] *[BZ, RZ]	58 [25 - 76] *[IZ, RZ]	104[93 - 119] *[IZ, RZ]	ns
	Lean	16 [1 - 25] *[BZ, RZ]	39 [23 - 72] *[IZ, RZ]	108 [98 - 119] *[IZ, RZ]	
HAD (µmol/g/min)	Obese	10 [1 - 34] *[BZ, RZ]	47 [17 - 62] *[IZ, RZ]	94 [80 - 103] *[IZ, RZ]	ns
	Lean	10 [1 - 15] *[BZ, RZ]	26 [14 - 52] *[IZ, RZ]	96 [83 - 102] *[IZ, RZ]	

Data presented as median and interquartile range [IQR]. CS; Citrate synthase activity. HAD; β-hydroxy-acyl-CoA dehydrogenase activity. *$P < .05$; indicates significant differences to other zones indicated by their abbreviation (IZ, BZ, RZ). #$P < .05$; indicates significant group difference between lean and obese animals for the given zone.
